# Surface reinforcement of rice straw fibers for epoxy composites by ultrasonic, plasma, and sodium carbonate-assisted hybrid treatments

**DOI:** 10.1039/d5ra08748a

**Published:** 2026-01-12

**Authors:** Harianingsih, Sivasubramanian Palanisamy, Deni Fajar Fitriyana, Januar Parlaungan Siregar, Nur Qudus, Saleh A. Alfarraj, Sulaiman Ali Alharbi, Mohamed Abbas, Shaeen Kalathil, Mezigebu Belay

**Affiliations:** a Department of Chemical Engineering, Faculty of Engineering, Universitas Negeri Semarang Sekaran Gunungpati Semarang Central Java 50229 Indonesia harianingsih@mail.unnes.ac.id; b Department of Mechanical Engineering, School of Engineering, Mohan Babu University Tirupati Andhra Pradesh 517102 India sivaresearch948@gmail.com; c Department of Mechanical Engineering, Faculty of Engineering, Universitas Negeri Semarang Sekaran Gunungpati Semarang Central Java 50229 Indonesia; d Faculty of Mechanical and Automotive Engineering Technology, Universiti Malaysia Pahang Al-Sultan Abdullah (UMPSA) 26600 Pekan Malaysia; e Automotive Engineering Center (AEC), Universiti Malaysia Pahang Al-Sultan Abdullah (UMPSA) 26600 Pekan Malaysia; f Department of Civil Engineering, Faculty of Engineering, Universitas Negeri Semarang Sekaran Gunungpati Semarang Central Java 50229 Indonesia; g Department of Zoology, College of Science, King Saud University P.O. Box 2455 Riyadh 11451 Saudi Arabia; h Department of Botany and Microbiology, College of Science, King Saud University P.O. Box.2455 Riyadh 11451 Saudi Arabia; i Electrical Engineering Department, College of Engineering, King Khalid University Abha 61421 Saudi Arabia; j Department of Condensed Matter Physics, Saveetha School of Engineering, Saveetha Institute of Medical and Technical Sciences (SIMATS) Thandalam Chennai India; k Department of Electrical Engineering, College of Engineering, Princess Nourah bint Abdulrahman University P.O. Box 84428 Riyadh 11671 Saudi Arabia; l Department of Metallurgical and Materials Engineering, College of Engineering, Ethiopian Defence University Bishoftu 1041 Ethiopia mezgebubelay@etdu.edu.et

## Abstract

Rice straw represents a plentiful agricultural by-product that remains largely underexploited, particularly for composite reinforcement, due to poor fiber-matrix interactions and its high amorphous fraction. In this study, environmentally benign surface modification strategies were explored to ensure better mutual performance of rice-straw grains with an epoxy pattern. Four activation approaches were evaluated: ultrasonic treatment (P1), ultrasonic assisted with sodium carbonate (P2), plasma exposure (P3), and a combined Na_2_CO_3_-plasma sequence (P4). Fibers were processed using 5% w/v Na_2_CO_3_ solution and low-pressure plasma at 13.56 MHz, followed by fabrication of epoxy composites. The materials were examined through several analytical methods, including flexural evaluation (ASTM D790), FTIR spectroscopy, SEM-EDX imaging, XRD diffraction, TGA/dTG thermal analysis, and BET surface analysis. An overall enhancement in mechanical characteristics was detected as the degree of treatment increased. Sample P1 had a flexural durability of approximately 109.1 MPa, while sample P4 showed elasticity at 162.0 MPA and the range of modalities in terms of their articulation was expanded by 5.625 GPa (passive modulus) from 3.709 GPa when tested against other methods. SEM micrographs revealed remarkable surface alterations, such as a 131% rise in micro-texture roughness (from 0.344 to 0.796), resin deposition reaching 90.8%, a 72% decline in pore or void fraction (from 3.319% to 0.917%), and an 84% reduction in silica or ash residues. XRD profiles showed more pronounced cellulose-I reflections at 15.7°, 22.6°, and 34.6°, alongside the suppression of the amorphous halo (18–20°), signifying increased crystallinity, particularly in P4 fibers. TGA results demonstrated reduced char residue and higher, sharper Tmax peaks, confirming improved thermal stability. Among the treatments, the Na_2_CO_3_-assisted plasma approach (P4) provided the most substantial enhancement, offering a scalable and sustainable method to upgrade rice-straw fibers for structural composite applications.

## Abstract 2

Surface-treated rice straw fibers improve epoxy composite strength and interfacial properties.

## Graphical abstract

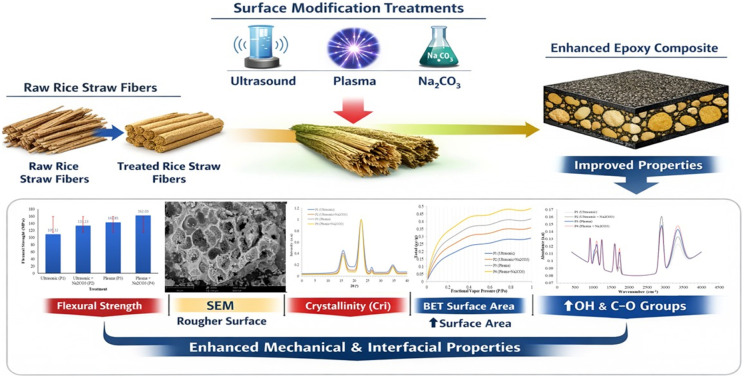

## 1 Introductions

Indonesia ranks as the fourth-largest rice producer globally. According to national statistics, rice cultivation in Indonesia is projected to cover approximately 10.20 million hectares in 2023, yielding around 30.90 million tons of rice. This substantial output generates various agricultural by-products, such as paddy straw, husk, ash, bran, and cracked grains.^1^ Paddy straw, the primary residue from rice farming, is produced at an estimated annual rate of nearly 50 million tons. However, its utilization remains limited, with only about 20% being used in industries like paper production, fertilizers, animal feed, and bioenergy.^2^ The remaining straw is often left unused or burned, leading to serious environmental issues, including air pollution and higher CO_2_ emissions, which contribute to global warming.^3^

Several methods have been explored to enhance the value of rice straw, including its conversion into bioenergy^4^ and its use as a lignocellulosic reinforcement in composite materials.^5^ Despite these efforts, further advancements remain necessary, especially in improving the interfacial behavior between the rice straw fibers and the polymer matrix. This interfacial bonding is crucial for determining the mechanical performance of the resulting composites. Rice straw fibers naturally contain surface waxes, moisture, and abundant hydroxyl groups, which impede effective adhesion with polymer matrices, thereby reducing composite strength.

Various modification techniques are employed to improve fiber-matrix bonding, including chemical treatments, physical modifications, and biological methods. Common chemical modifications include alkaline treatments and acid hydrolysis.^6^ Physical approaches consist of plasma treatment, ultrasonic irradiation, and steam explosion. Biological modifications typically rely on enzymatic processes or fermentation systems.^7^ Although chemical methods prove effective, they often present challenges related to toxicity, environmental risks, and increased costs. On the other hand, biological treatments, while more environmentally friendly, tend to have lower stability, limited control, and longer processing times.^8^ Therefore, the development of rice straw-based composites still requires more efficient, rapid, and eco-friendly modification strategies to ensure sustainability.^9^

In this study, a hybrid surface-modification approach combining chemical and physical treatments was used to enhance the interfacial interaction between rice-straw fibers and the polymer matrix. The method involved both ultrasonic and plasma activation in an alkaline medium derived from sodium carbonate. Ultrasonic treatment generates high-frequency acoustic waves that create bubbles in the liquid phase.^10^ These bubbles induce cavitation effects, generating microscopic perturbations on the fiber surface. This results in increased surface roughness and a larger interaction area between the fiber and the polymer, improving interfacial bonding. Additionally, ultrasonic treatment helps to remove surface wax layers, exposing more reactive sites for polymer attachment.^11^

The cavitation effect during ultrasonic processing physically alters the fiber surface, enhancing its affinity for the polymer and facilitating stronger anchoring within the matrix.^12^ The localized surface wear at the fiber-matrix boundary increases the interfacial zone, promoting better adhesion. Furthermore, ultrasonic agitation facilitates the penetration of alkaline agents into the fiber, generating new reactive functional groups and enabling *in situ* chemical modifications. The transmitted acoustic power also contributes to the partial breakdown of hydrogen bonding networks and the rearrangement of cellulose microfibrils, improving both the fiber surface topology and its interfacial behavior in composite materials.^13^

Plasma treatment is an eco-friendly surface modification technique that induces controlled changes to the surface characteristics of materials without altering their main components. This process enhances fiber wettability and promotes stronger interfacial bonding with the matrix.^14^ In plasma environments, energetic electrons ionize gas molecules to form reactive radicals, which initiate surface activation and plasma-induced polymer deposition, resulting in a thin, uniform surface layer. These alterations improve wettability, facilitate efficient stress transfer, and enhance interfacial properties in composites. Both acoustic-assisted activation and plasma exposure effectively remove wax layers and surface impurities while inducing micro-etching, which increases surface roughness. These synergistic modifications strengthen the adhesive interaction and structural integration between fibers and the polymer matrix, improving overall interfacial cohesion.^15^

Previous studies have reported improved bonding strength in wheat-straw-based composites following plasma treatment. These findings demonstrate that cold plasma is a promising, clean, and environmentally friendly approach for enhancing fiber-matrix adhesion in polymer composite systems. While there is growing interest in natural-fiber reinforcement, research on the surface modification of rice straw through ultrasonic treatment combined with alkaline salts or plasma processes is still limited, particularly for composite applications.^16^ Alkaline treatments are widely used due to their cost-effectiveness, ability to penetrate the fiber structure easily, and efficiency in exposing functional groups by removing lignin, hemicellulose, and waxy components. However, improvements from alkaline treatments alone often fall short compared to those achieved through ultrasonic or plasma-based methods.^17^ In response to these limitations, a hybrid modification approach combining Na_2_CO_3_ alkalization with physical treatment is employed in this study. This integrated method aims to enhance the mechanical performance of the composites, increase cellulose crystallinity while reducing amorphous components, and reinforce hydrogen bonding within cellulose structures. Consequently, the interfacial compatibility between fiber and matrix in polymer composites is expected to improve significantly.^18^

Wulandari *et al.* (2025) reported that alkaline treatment effectively removes non-cellulosic constituents, increases cellulose content, and introduces more accessible surface-bound hydroxyl groups, all of which improve interaction with polymer matrices and enhance load-bearing efficiency.^19^ The removal of non-cellulosic components exposes the underlying fibrillar morphology, increasing the available surface area for matrix interaction, which strengthens the composite material.^20^ Although there is substantial research on rice-straw-based composites, studies focusing on enhancing the performance of physical pretreatments through alkaline modification remain limited. Specifically, comprehensive investigations into the combined effects of physical processes, such as ultrasonication or plasma treatment, with mild alkaline solutions in modifying surface chemistry, morphology, and interfacial bonding have not been sufficiently explored.^21^

This research contributes by developing a hybrid modification strategy that integrates Na_2_CO_3_ treatment with ultrasonication and plasma processes to optimize the surface properties of rice straw fibers for composite applications. This approach provides a cleaner, more cost-effective alternative to traditional chemical modification techniques. Beyond improving the reinforcing potential of rice straw, this method supports the sustainable utilization of agricultural biomass and contributes to the circular economy by converting agricultural waste into valuable resources.^22^

This research aims to evaluate and compare various surface-modification techniques applied to rice straw fibers. Plasma treatment and ultrasonic treatment, in combination with the eco-friendly alkaline agent Na_2_CO_3_, will be assessed using a range of characterization methods. These include surface morphology and silica distribution analysis *via* SEM, complemented by elemental identification through EDX, as well as thermal stability assessment through thermogravimetric analysis (TGA). The rice straw samples showing the most favorable structural and surface improvements will be incorporated into polymer composites, and their mechanical performance will be evaluated through tensile strength testing. These environmentally sustainable modification strategies are expected to significantly enhance fiber-matrix interaction, thereby improving the strength and durability of the resulting composites.

## 2 Methodology

### 2.1 Materials

Cellulosic fibers obtained from rice straw used in this work were collected from Tasikmalaya, Indonesia. After drying, the straw was chopped into short fragments, milled into finer particles, and screened using a 250 µm sieve to obtain uniform fiber size. The fraction passing through the sieve was subsequently dried in an oven at 80 °C for 24 hours to guarantee complete removal water content prior to modification. A sodium carbonate solution Merck 106392.0500 with a concentration of 5% w/v was prepared for alkaline treatment. The composite matrix consisted of an epoxy resin system cured with EPH 555 hardener at a mixing ratio of 2 : 1.^23^

### 2.2 Ultrasonic treatment

In this experiment, rice straw fibers were treated using various methods to improve their flexural strength. For ultrasonic treatment (P1 and P2), the rice straw fibers were dissolved in a 5% Na_2_CO_3_ (w/v) solution with a fiber-to-solution ratio of 1 gram of fiber per 50 mL of solution. The ultrasonic treatment was applied at a frequency of 30 kHz for a duration of 15 minutes with alternating pulses (10 seconds on, 10 seconds off). To prevent thermal and mechanical damage to the fibers, the temperature during the ultrasonic treatment was kept below 35 °C using an ice bath. After sonication, the fibers were heated to 60 °C for 60 minutes with gentle stirring to promote the saponification of waxes and pectin, as well as the removal of hemicellulose. For plasma treatment (P3 and P4), the pre-washed and sectioned rice straw fibers were immersed in a 5% (w/v) Na_2_CO_3_ solution with a fiber-to-solution ratio of 1 gram of fiber per 50 mL of solution. Plasma treatment was conducted at a frequency of 13.56 MHz with a power of 120 W, a pressure of 0.3 bar, and an airflow of 50 L min^−1^ for 6 minutes. The process temperature was controlled at 50 °C to minimize thermal damage to the fibers. Upon completion of the plasma treatment, the specimens were immediately placed in a desiccator to protect them from ambient humidity and particulate contamination. Each treatment stage was followed by three washing cycles until the filtrate reached a neutral pH (∼7), and the electrical conductivity of the rinse water indicated the removal of ionic residues. Finally, the treated fibers were dried in an oven at 70 °C for 18 hours and stored in a desiccator with a relative humidity of ≤30% before being used in the subsequent steps.^24^

### 2.3 Modification surface using plasma

Pre-washed and sectioned rice straw fibers were immersed in a 5% (w/v) Na_2_CO_3_ solution with a fiber-to-solution ratio of 1 g of fiber per 50 mL of solution. After soaking, the fibers were evenly placed on a sample holder to ensure uniform interaction with the plasma. The plasma treatment was performed under glow-discharge conditions at 13.56 MHz, with a power of 120 W, a chamber pressure of 0.3 bar, and an airflow rate of 50 L min^−1^ for 6 minutes. The process temperature was precisely maintained at 50 °C to minimize thermal damage to the fibers. Upon completion of the plasma treatment, the specimens were immediately transferred to a desiccator to protect them from ambient humidity and particulate contamination.^25^

### 2.4 Composite preparation

The processed rice-straw reinforcements, previously subjected to acoustic and plasma-based surface activation, were removed from the dry storage chamber and then oven-dried at 60 °C for 12 hours. Before composite formation, the mold cavity was pre-treated with a separating compound to prevent sticking during demoulding. The epoxy formulation, prepared at a 2 : 1 epoxy-to-hardener ratio, was mixed for 3 minutes at 50 rpm to ensure uniformity while avoiding excessive air entrapment, followed by vacuum degassing for 5 minutes to eliminate residual bubbles. The fibers were impregnated by evenly distributing the resin over the fiber layers, followed by vacuum bagging at approximately 0.8 bar to minimize void formation and control resin uptake. The composite was initially cured at at 25 °C for 24 hours and subsequently post-cured at 80 °C for 1 hour to promote optimal network formation. Once curing was complete, the laminate was removed from the mold and sectioned into test specimens according to the applicable standards.^26^

### 2.5 Analysis

A three-point bending method compliant with ASTM D790 was employed to measure the flexural strenght, where the test specimens had a span-to-depth ratio of 16 : 1, and their initial thickness and width were measured prior to testing. The movement rate of the testing head was calculated using the conventional equation to ensure the designated outer-layer strain rate. The bending strength and modulus values were derived from the corresponding load-displacement curves.^27^ Chemical changes on the fiber surface were analyzed *via* FTIR in the wavenumber interval of 4000–400 cm^−1^, spectral data were collected at a resolution of 4 cm^−1^ using 32–64 scans, subsequently subjected to baseline correction and normalization before identifying key absorption bands associated with modification-induced functional group transformations.^28^

The surface and interfacial characteristics of the fibers were analyzed using a scanning electron microscope (SEM) operated at an accelerating voltage of 5–10 kV. Prior to imaging, specimens were sputter-coated with a thin Au/Pd layer (5–10 nm). Images at multiple magnifications were obtained to assess surface purity, texture, resin infiltration, fracture mechanisms (fiber pull-out or breakage), and void formation. The SEM images obtained after treatment were analyzed using NanoMeasure to calculate the polymer-coated surface area. This process involves extracting and measuring the area covered by the polymer, and then determining the polymer coating percentage by calculating the ratio of the coated area to the total area of the image. Measurements were taken from three random locations to ensure accuracy and representativeness of the results. To assess the micro-scratches formed on the fiber surface, NanoMeasure was used to measure the depth and length of the scratches based on color gradient differences in the SEM images. Each scratch was measured at five random locations, providing insights into the abrasion level of the fiber surface after treatment.^29^

Elemental mapping through qualitative EDS was additionally performed when necessary to identify inorganic traces. X-ray diffraction (XRD) with Cu Kα radiation (*λ* = 1.5406 Å) was employed to evaluate the structural ordering of the samples across a 2*θ* range of 5–80°, using a 0.02° step size and 1–2 s counting time. Following background correction and slight smoothing, the main diffraction peaks were ordered, degree of crystallinity was determined where applicable, and band broadening was analyzed to estimate the crystal grain size and lattice imperfection. Regarding the CrI calculation, we have now included the Segal method and the relevant equation for CrI calculation in the revised manuscript. Specifically, CrI is calculated using the formula:^30^1
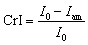
where *I*_0_ is the intensity of the crystalline 002 peak, and *I*_am_ is the intensity of the amorphous peak at approximately 18°.

Test were performed on thermal stability using TGA, a method that uses stale air to maintain temperature under nitrogen gas conditions with varying flow rates of 50 to 60 mL per minute. The test was carried out by heating the sample from 30 Celsius up to 800 Celsius at a rate of 10 Celsius per minute. The breakdown of hemicellulose, lignin, and resin was depicted as the temperatures at which the major breakage steps began (Tonset) and concluded (*A*_max_) on an independent thermogravimetric curve (dTG) chart. The amount of material left after heating was also measured between 700 and 800 Celsius. These results were compared across different treatments to see how surface modification affected thermal resistance and the amount of inorganic material present.^31^ The specific surface area and porous characteristics of the modified rice straw fibers and their composite materials were examined using nitrogen adsorption–desorption evaluation based on the Brunauer–Emmett–Teller (BET) theory. Before the analysis, the samples were pretreated by evacuating under reduced pressure at 80–120 °C for 6–12 hours, depending on their thermal resistance, to eliminate retained water and volatile residues. Nitrogen sorption isotherms were acquired at 77 K with a volumetric gas sorption apparatus, spanning relative pressure ratios (*P*/*P*_0_) from 0.01 to 0.99.^32^

## 3 Results

### 3.1 Flexural strenght performance

**Fig. 1:**
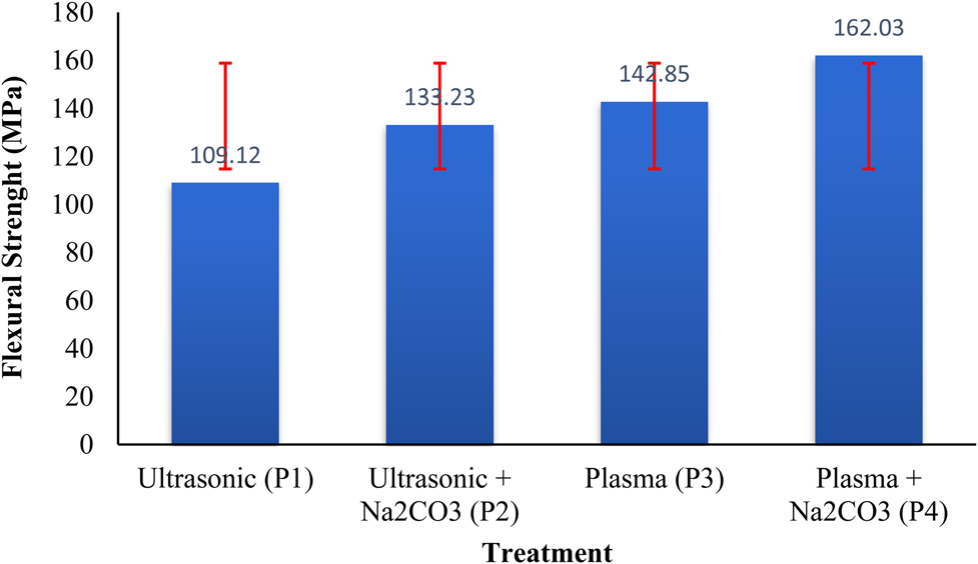
Effect of surface treatment method on flexural strenght.

A progressive improvement in bending characteristics was observed as the intensity of surface treatment increased. The results shown in Fig. 1 indicate that incorporating Na_2_CO_3_ during ultrasonic or plasma treatments led to the greatest enhancement. The mean flexural strength increased from 109.1 MPa under ultrasonic treatment to 133.2 MPa when combined with Na_2_CO_3._ Plasma treatment further increased the value to 142.9 MPa, reaching its peak at 162.0 MPa when Na_2_CO_3_ was incorporated. These results demonstrate a synergistic improvement in the interfacial bonding between the fibers and matrix.^33^ The alkaline treatment dissolves non-cellulosic carbohydrates, phenolic compounds, waxy layers, and oily residues, while also reducing silica content. This results in greater exposure of hydroxyl groups and enhanced surface roughness, which improves the wettability and continuity of the fibers with the matrix.^34^ The treatment with Na_2_CO_3_ induces ester saponification on the fiber surface, which aids in the removal of lignin and hemicellulose, while enhancing the exposure of hydroxyl (–OH) groups on the fiber surface. Additionally, Na_2_CO_3_ acts as a partial delignification agent, reducing the amorphous components of the fiber and improving its rigidity and adhesion to the polymer matrix.^35^ Plasma treatment further enhances the fiber-matrix interaction by generating polar oxygen-based functional groups and forming a thin, uniform layer between the fibers and surrounding material. This layer improves fiber adhesion, reduces the formation of air pockets, and strengthens the bond between the fibers and matrix.^36^ Plasma treatment induces surface oxidation and the formation of free radicals, which enhance the fiber surface's polarity. This oxidation improves the fiber's ability to interact with the polymer matrix, strengthening the interfacial bond and accelerating the integration of the fiber into the matrix.^37^ These improvements facilitate better stress transfer across the fiber-matrix interface, leading to increased bending resistance and greater structural stiffness. These findings align with previous studies on natural fiber composites treated with alkali or plasma.^38^

**Fig. 2:**
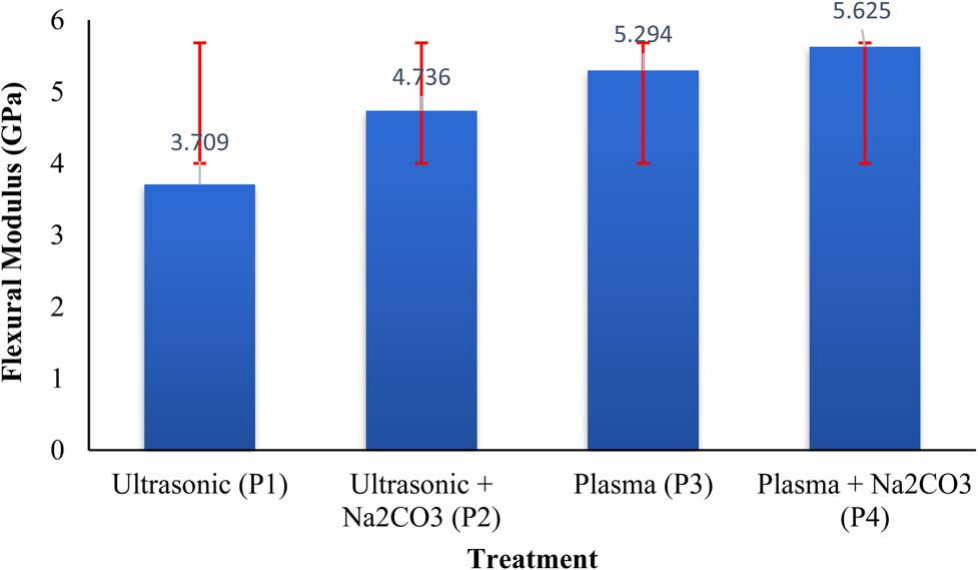
Effect of surface activation technique on flexural modulus.

As shown in Fig. 2, the flexural modulus consistently increased across all treatments. The GPa values were 3.709 GPa for ultrasonic treatment, 4.736 GPa for ultrasonic + Na_2_CO_3_, 5.294 GPa for plasma, and 5.625 GPa for plasma + Na_2_CO_3_. Compared to P1, the stiffness increased by 27.7% for P2, 42.7% for P3, and 51.7% for P4. Additionally, P4 showed a 6.3% improvement over P3 and an 18.8% increase compared to ultrasonic treatment with Na_2_CO_3_. These findings align with those of Siddiqui *et al.* (2023), who observed that Na_2_CO_3_ treatment after plasma activation enhances surface polarity and energy, leading to better bonding and mechanical adhesion by removing plant materials and increasing surface roughness.^39^ The removal of non-crystalline components such as lignin and hemicellulose exposes the more rigid crystalline cellulose, contributing to the improved flexural modulus.^40^ The combined effects of plasma exposure and alkaline treatment amplify surface roughness and chemical reactivity, facilitating efficient stress transfer and enhancing composite cohesion.^41^

**Fig. 3:**
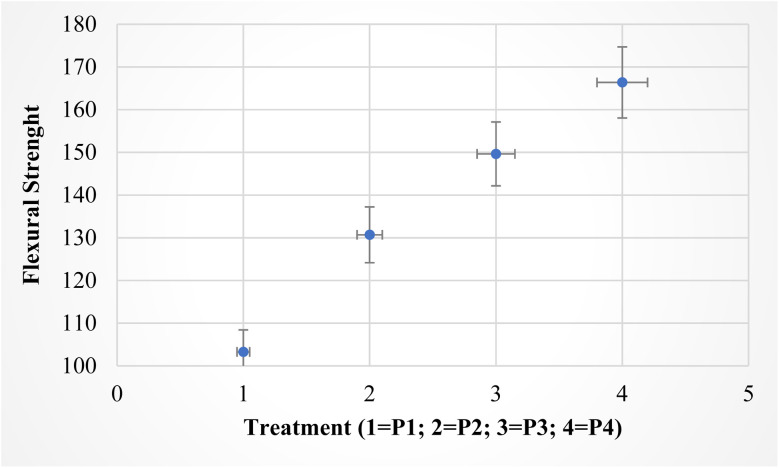
ANOVA analysis for flexural strenght.

The boxplot illustrating the results of the ANOVA for flexural strength across the four treatments (P1, P2, P3, and P4). The plot Fig. 3 shows a clear visual representation of the differences in flexural strength between the treatments.

The ANOVA results in Fig. 3 indicate a significant difference in the flexural strength across the four treatments (P1, P2, P3, P4) with an F-statistic of 356.67 and a *p*-value of 2.11 × 10^−22^ which is well below the 0.05 threshold. This shows that the differences observed in flexural strength between the treatments are statistically significant. P1 demonstrates the lowest average flexural strength, implying that this treatment did not notably enhance the fiber's properties when compared to the other treatments.^34^ In contrast, P2 shows an improvement in flexural strength, which suggests that the incorporation of Na_2_CO_3_ increases the fiber's mechanical properties, likely due to enhanced surface reactivity and the removal of non-cellulosic substances.^35^ The flexural strength continues to improve with P3, indicating that plasma treatment effectively modifies the surface chemistry of rice straw fibers, thereby enhancing their interaction with the polymer matrix.^36^ The greatest flexural strength is observed in P4, highlighting the synergistic effect of combining both plasma treatment and Na_2_CO_3_. This combination likely promotes better surface activation and exposes more functional groups, improving the fiber-matrix adhesion.^37^ The error bars, which represent the standard deviation, show less variability in P4, indicating more consistent results, whereas P1 has larger error bars, signifying more fluctuation in the data. This result according with Siddiqui *et al.* (2023), the synergy between plasma and Na_2_CO_3_ facilitates stronger surface activation, enhancing the exposure of functional groups such as hydroxyl (–OH) and carbonyl (C=O), which improves the adhesion of fibers to the polymer matrix.^39^ This finding highlights that the combination of physical and chemical treatments is significantly superior to separate treatments, marking an important new discovery in the development of natural fiber-based composites.^25^

### 3.2 Interfacially-active moieties

**Fig. 4:**
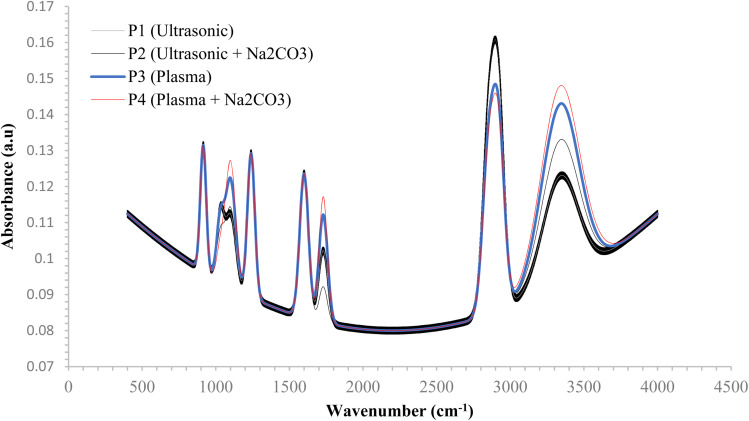
Spectral analysis of composite reinforced with rice straw fibers.

The infrared spectrum shown in Fig. 4 highlights key peaks: O–H stretching between 3300 and 3400 cm^−1^, aliphatic C–H stretching at 2850 and 2920 cm^−1^, a C=O bond around 1720 cm^−1^, C–O binding at approximately 1050 cm^−1^, and epoxide ring vibration near 915 cm^−1^. The C=O and C–H bands, both present in the epoxy matrix but not significantly different, suggest interactions between the cellulose hydroxyl groups and the polymer matrix. As noted by Badagliacco *et al.* (2021), ultrasonic processing reduces hemicellulose and lignin signals, evidenced by the decreased absorption near 1737 cm^−1^, typically associated with hemicellulose/lignin carbonyl groups. This indicates partial removal of amorphous components and increased exposure of cellulose –OH sites.^42^ Ultrasonication modifies the chemical composition of the fiber's surface without altering its fundamental structure.^43^ The absorption signals in the 1000–1500 cm^−1^ range confirm that key natural fiber components, including cellulose, hemicellulose, lignin, and pectin, remain intact after treatment.^44^ Overall, the spectral changes indicate a mild surface cleaning effect, characterized by a slight reduction in wax and hemicellulose features and a sharper –OH band, reflecting partial surface opening rather than full activation. These findings are consistent with Parthasarathy *et al.* (2023), who observed similar behavior in gentle alkaline conditioning, where non-cellulosic materials are reduced, improving surface accessibility. Specifically, the reduced C=O peak near 1732 cm^−1^ signals the removal of carbonyl groups linked to hemicellulose and lignin, supporting the reduction of surface waxes and leached hemicellulose, as also indicated by the weakened carbonyl band around 1712 cm^−1^.^45^

The application of ultrasonic energy combined with Na_2_CO_3_ induces a more pronounced restructuring of the fiber's surface chemistry. Vibrational bands associated with hemicellulose- and pectin-derived esters near 1730 cm^−1^, along with C–O ester features at 1240–1260 cm^−1^, experience significant attenuation, while the aliphatic C–H peaks around 2920/2850 cm^−1^ decrease, indicating effective removal of surface waxes. Additionally, an enhancement in the O–H stretching region (3300–3400 cm^−1^) reflects increased exposure of hydroxyl groups and improved hydrophilicity. Some silica-based ash may also have been removed, as evidenced by the weaker absorption in the Si–O/Si–OH regions between 1000–1100 cm^−1^ and 450 cm^−1^. Furthermore, the bands in the range of 1609 to 1550 cm^−1^, typically associated with the vibrations of lignin's aromatic structures, suggest significant breakdown of lignin through the combined ultrasonic and alkaline treatment.^46^ The Na_2_CO_3_ modification promotes substantial removal of hemicellulose and lignin, facilitates saponification of surface waxes, and reduces the fiber's contact angle by generating additional accessible hydroxyl groups.^47^ This increased availability of –OH groups enhances interfacial adhesion *via* hydrogen bonding and helps minimize void formation within the composite matrix.^48^

Plasma treatment introduces additional oxygen-containing functional groups, as indicated by enhanced absorption bands corresponding to carbonyl stretching around 1720 cm^−1^, ether or alcohol linkages approximately 1100 cm^−1^, and hydroxyl vibrations in broad region near 3400 cm^−1^, while reducing the intensity of C–H signals in 2920/2850 cm^−1^. The increased hydroxyl group content in the P3 and P4 samples improves their bending strength through enhanced interaction with the epoxy and hardener. The sequential plasma-Na_2_CO_3_ treatment (P4) produces the most chemically active and contaminant-free interface, as reflected by the maximized polar functional bands (O–H, C=O, C–O) and the suppression of silica-related Si–O/Si–O–Si features.^49^ These findings align with previous studies, which indicate that increased surface polarity, thermal stability, and hydrolytic resistance contribute to more effective stress transfer and reduced susceptibility to moisture-induced degradation.^50^

### 3.3 Surface topography and microstructural roughness examined using scanning electron microscopy

The surface alterations were investigated through SEM imaging of treatment at interface, with a focus on surface features and the distribution of empty spaces.^51^

**Fig. 5:**
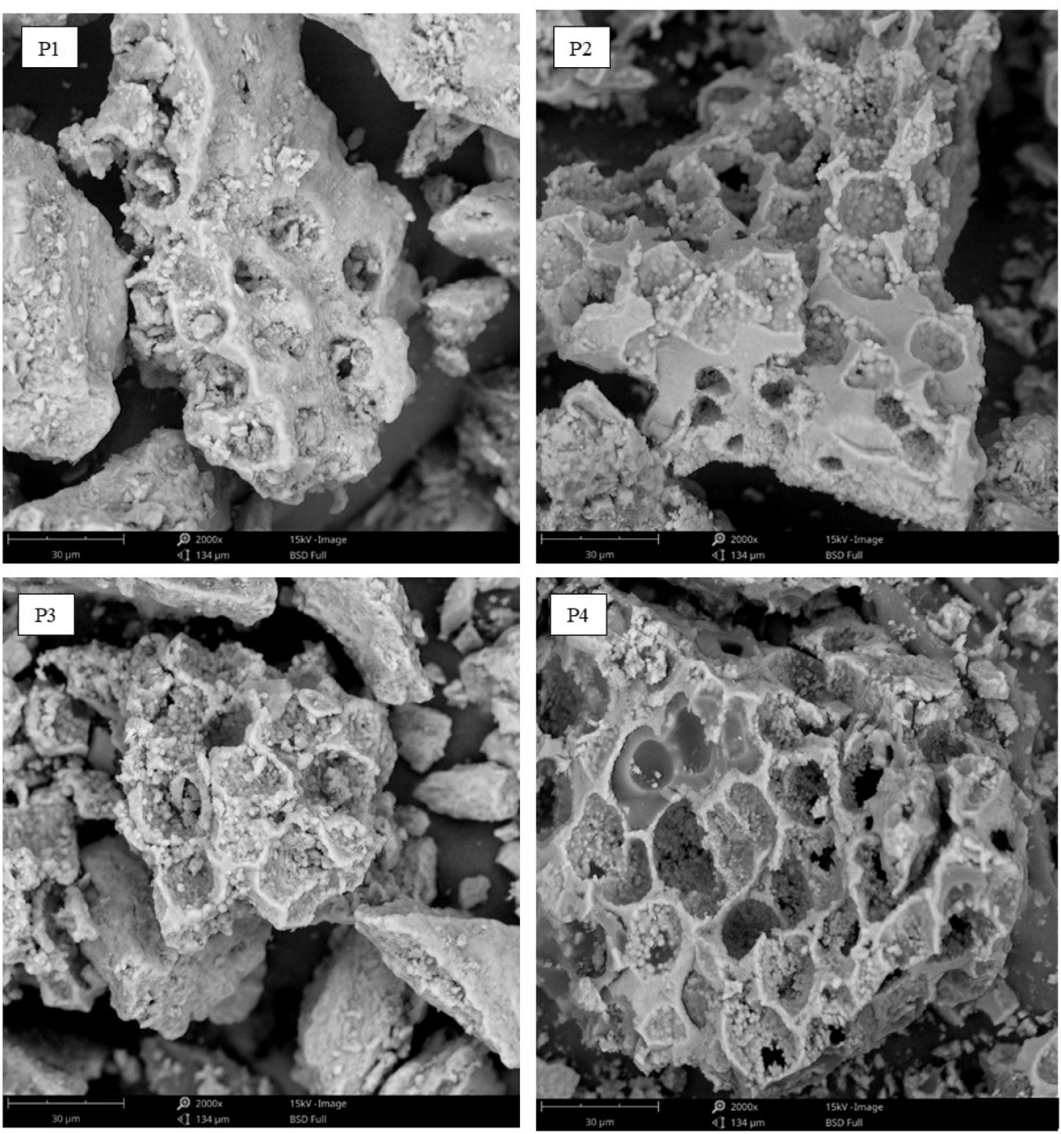
Surface area characterization of P1 through P4.

As illustrated in Fig. 5 and Table 1, a concurrent improvement is observed from ultrasonic activation to plasma soda activation. Surface roughness increases significantly, with P4 (0.796) showing a ∼131% increase compared to P1 (0.344), which is attributed to plasma etching combined with Na_2_CO_3_-induced surface opening. Resin coverage also improves, rising from 73.1% in P1 to 90.8% in P4, reflecting a 24% increase and indicating more consistent wetting and resin penetration. The void fraction decreases notably, from 3.319% in P1 to 0.917% in P4, representing a 7.43% reduction in internal defects. Additionally, silica particle density drops significantly, from 121 to 19 particles per mm^2^ (∼84% decrease), confirming a cleaner fiber surface and better interfacial compatibility in the P4 treatment. The pull-out lengths for P1 and P4 differ, with P2 showing a 30% increase and P3 showing a 7.43% increase, while the other treatments exhibit slight variations in debond lengths. These pull-out data suggest stronger bonding between the fibers and the matrix.^52^

**Table 1:** Evaluation of interfacial morphology in surface-modified epoxy composites reinforced with rice straw fibers *via* SEM

Measured	Analysis			
	P1 (ultrasonic activation)	P2 (ultrasonic + soda activation)	P3 (plasma activation)	P4 (plasma-soda activation)
Micro-scratch abrasion (a.u.)	0.344	0.558	0.722	0.796
Polymer coating extent (%)	73.083	83.371	86.882	90.82
Porosity percentage (%)	3.319	2.338	1.501	0.917
Inorganic particle concentration (count/mm^2^)	121.029	59.255	44.717	19.408
Fiber detachment length (µm)	45.07	30.413	23.081	15.775
Interface delamination length (%)	30.02	15.756	10.226	7.432
Mean fiber thickness (µm)	15.306	15.252	14.778	15.035
Pore entrance width at interface (nm)	195.387	186.592	143.616	118.55

Regarding pore size, P1 (195 nanometers) and P4 (119 nanometers) represent the most common yet least abundant sizes, indicating more compact interfaces, fewer voids, and reduced cracking.^53^ The fiber diameter remains consistent at 15 µm across all treatments from P1 to P4, suggesting that Na_2_CO_3_ modification contributes to maintaining fiber dimensional stability. The results indicate that P4 exhibits the best performance, followed by P3, P2, and P1. Plasma treatment is effective in altering the surface properties of rice straw fibers used in composite materials, enhancing surface polarity and energy, which improves fiber adhesion to the matrix. When combined with Na_2_CO_3_, the improvement is even more pronounced.^54^ Na_2_CO_3_ removes softer fiber components, such as lignin and hemicellulose, and increases the number of –OH groups on the surface. These –OH groups form hydrogen bonds that enhance the interaction between the fibers and the matrix. Consistent with Sanfilippo *et al.* (2024), this study demonstrates that initial alkaline treatment followed by plasma treatment improves the adhesion between natural fibers and the matrix, resulting in stronger composite materials.^55^

### 3.4 Improvement of crystalline structure in rice straw fibers

Rice straw fibers underwent structural changes following various treatments, which were studied using X-ray diffraction (XRD). A review was conducted to determine if the structure of rice straw composites improved and weakened, while also measuring the strength (and wearability) rating.^56^

**Fig. 6:**
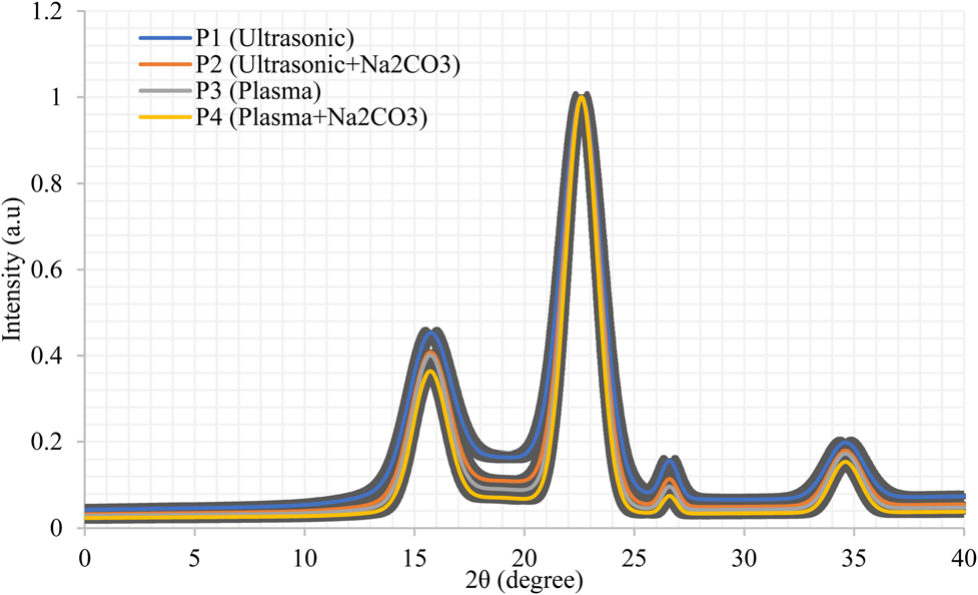
Comparative XRD analysis of modified rice straw fibers.

Fig. 6 illustrated the Bragg reflections at 2*θ* values of 15.7°, 22.6°, and 34.6°, characteristic of a monoclinic cellulose structure. The broad scattering bands near 18° and 20° correspond to amorphous scattering from hemicellulose fractions and the lignocellulosic matrix.^57^ Across the P1–P4 treatments, the progressive sharpening of crystalline peaks and the reduction in intensity of non-crystalline regions indicate an increase in the crystallinity index (CrI). Higher CrI values are linked to enhanced fiber rigidity, improved structural stability, and reduced hygroscopicity.^58^ The final P4 treatment results in a more uniform and well-aligned cellulose microfibrillar network, minimizing microstructural degradation.^59^ Additionally, Na_2_CO_3_-mediated delignification effectively removes hemicellulose components and breaks lignin–cellulose linkages, thereby preserving the crystalline regions of cellulose while reducing steric constraints.^60^

Plasma treatment enhances crystallinity by etching the surface and opening the microfibrillar structure.^61^ The combination of these treatments produces a synergistic effect, with P4 exhibiting the highest CrI and the sharpest diffraction peaks among all samples. Increased cellulose crystallinity (CrI) is associated with improved fiber properties, including higher tensile strength, greater flexural rigidity, stronger bonding between fibers and the surrounding matrix, reduced moisture absorption, and better heat resistance.^62^ As such, the P4 treatment emerges as the optimal method for enhancing the performance of rice straw fibers. These results align with previous studies, which indicate that the removal of softer plant components, primarily hemicellulose and lignin, leads to an increase in CrI.^63^ Additionally, Santhosh *et al.* (2024) demonstrated that the combined use of plasma treatment and Na_2_CO_3_ delignification enhances tensile performance and fiber-matrix interfacial cohesion, improving stress transfer from the epoxy matrix to the fibers. This improved stress conduction is critical for achieving higher mechanical performance in fiber-reinforced systems.^64^

### 3.5 Heat-induced thermal breakdown behavior of alkali- and plasma-treated rice straw fibers

**Fig. 7:**
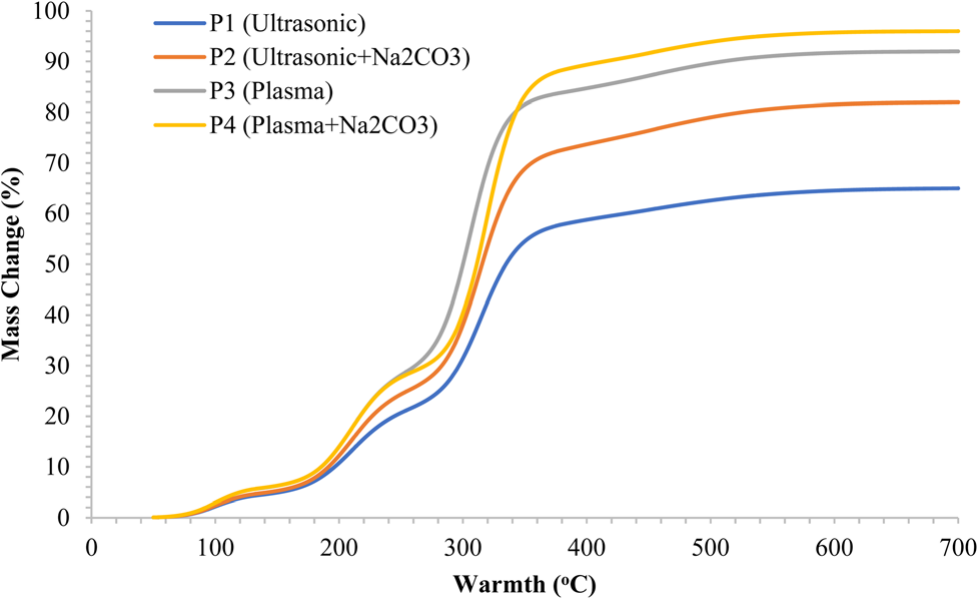
TGA curves of rice straw fibers under different surface treatments.

Fig. 7 illustrates the different stages of degradation as observed through thermogravimetric analysis (TGA). Moisture is lost up to 150 °C, followed by the breakdown of hemicellulose between 200 and 260 °C. Cellulose degradation occurs between 280 and 360 °C, while lignin decomposes above 400 °C, indicating the rupture of bonds between lignin and cellulose. After Na_2_CO_3_ treatment, the remaining mass is reduced, and the curves for the P2 and P4 samples become steeper compared to the untreated samples, suggesting the removal of amorphous biopolymers, which results in easier breakdown upon heating. TGA measures the ability of materials to convert to carbon and their thermal stability.^65^ A significant mass loss at lower temperatures indicates poor heat resistance. The stepwise mass decline corresponds to the evaporation of moisture and the decomposition of hemicellulose, which accounts for approximately 12% of the total weight, while cellulose constitutes around 60%, with the remaining mass being composed of lignin and residual char.^66^

Fig. 7 presents the mass-change thermograms, where P4 leaves less char compared to P1, P2, and P3. Plasma treatment combined with Na_2_CO_3_ effectively disrupts the bonding between lignin and cellulose, facilitating the breakdown of these bonds. As a result, P4 decomposes more cleanly and uniformly, while P1 exhibits a broader, less distinct breakdown pattern and retains more residue. The reduced residue in P4 indicates a higher purity of cellulose.^67^

**Fig. 8:**
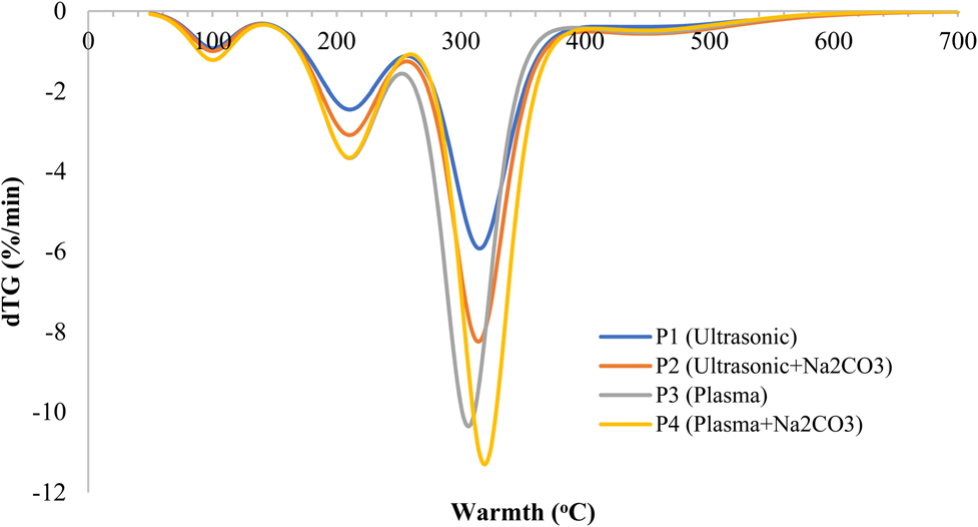
dTG traces showing the peak degradation temperature of rice straw fibers.

Fig. 8 presents the dTG curves, which depict the rate of mass loss across temperatures and provide insights into the degradation kinetics for each treatment. Four distinct dTG profiles correspond to the four sample treatments, illustrating the typical stages of plant material breakdown, such as for rice straw, when treated with ultrasound, plasma, or a combination with sodium carbonate. These stages include drying between 50 and 120 °C, hemicellulose decomposition between 220 and 380 °C, and lignin breakdown with char formation above 400 °C. P1 exhibits a main peak at around 347 °C with a mass loss rate of −8.1% per minute. P2 shows a peak at approximately 357 °C, with a higher rate of −9.0% per minute, suggesting a faster breakdown and higher cellulose content. P3 demonstrates greater thermal stability, with a peak at 361 °C and a smaller shoulder between 250–300 °C, indicating less hemicellulose.^68^ P4 shows the best thermal performance, with a peak at 368 °C and the highest mass loss rate of −10.8% per minute. The sharper peak suggests a more uniform and efficient breakdown, higher structural order, and increased cellulose purity due to hemicellulose removal. These findings align with previous studies, indicating that P4 exhibits the most robust degradation, reflecting improved stability in the rice straw-derived fiber structure.^69^ The sharp peak also indicates that cellulose decomposes more rapidly and selectively when the structure is simplified by the removal of hemicellulose and lignin. Plasma treatment aids by etching the surface, breaking chemical bonds, and increasing material flexibility, which promotes more uniform breakdown. In contrast, P1 shows a lower peak temperature and broader peak, indicating slower breakdown and a less uniform structure.^70^

The findings align with previous research, which has shown that plasma treatments generate reactive oxygen radicals, such as O and OH. These radicals attack the aromatic structures in lignin, breaking the β–O–4 bonds and accelerating lignin degradation. Concurrently, Na_2_CO_3_ induces saponification of the ester groups in hemicellulose, releasing acetyl units and reducing the amount of amorphous material. This leads to a more focused dTG peak during cellulose pyrolysis. Studies also suggest that when the dTG peak shifts to higher temperatures and becomes sharper, the activation energy increases, indicating enhanced crystallinity of cellulose.^68^ The sharper dTG peaks and higher crystallinity observed in this study are consistent with the findings of Rao *et al.* (2024), who demonstrated that increased crystallinity (CrI) accelerates degradation and minimizes the influence of amorphous regions. From a thermal perspective, more controlled high-temperature degradation improves interfacial stability in the composite, reducing the occurrence of thermal contraction, porosity, and interfacial degradation during matrix carbonization. Furthermore, uniform plasma treatment enhances fiber-matrix compatibility, resulting in consistent interfacial cohesion.^71^

### 3.6 BET adsorption–desorption assessment of chemically treated rice straw fiber composites

**Fig. 9:**
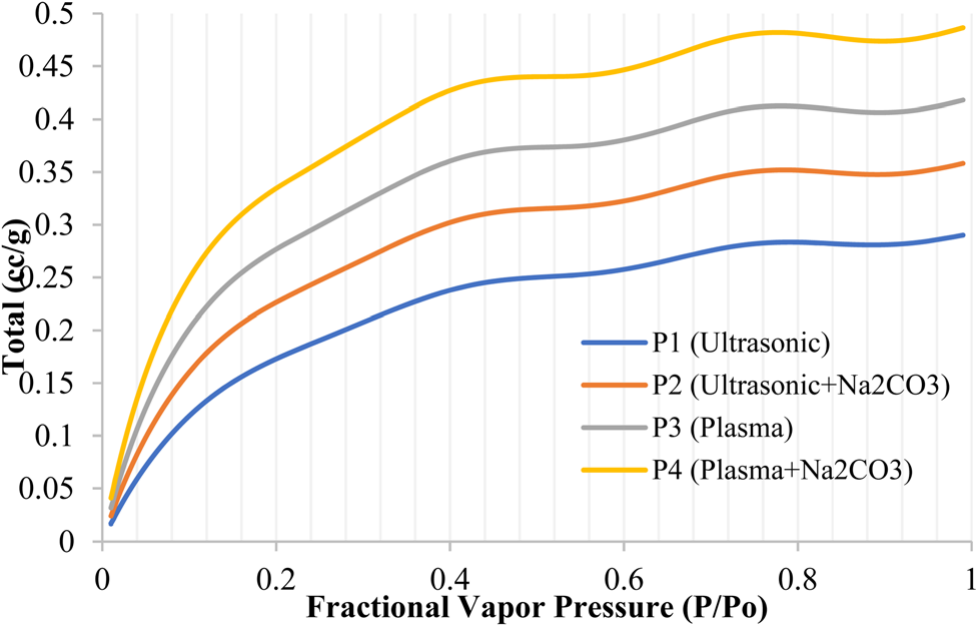
Characterization of modified rice straw fiber composites *via* nitrogen digestion.

In Fig. 9, the distribution of nitrogen adsorption curves indicates medium-sized pores. The BET surface area values for each sample are as follows: P1 = 120 m^2^ g^−1^, P2 = 135 m^2^ g^−1^, P3 = 145 m^2^ g^−1^, and P4 = 160 m^2^ g^−1^, demonstrating an increase in surface area with successive treatments. When the pressure ratio (*P*/*P*_0_) is about 0.99, which indicates the total pore volume, the values go in this order: P4 is the highest, followed by P3, P2, and P1. The adsorption data indicate a decreasing trend in specific volume from sample 1 to sample 4, consistent across all tested pressures, suggesting reduced porosity with successive treatments. At a pressure ratio of 0.10, which relates to very small pores and surface areas, the amounts absorbed are as follows: 0.22, 0.20, 0.16, and 0.12 cc g^−1^, in the same order. When the pressure ratio is 0.30, capillary condensation in medium pores begins, and the values are 0.40, 0.34, 0.27, and 0.22 cc g^−1^. At higher pressures, such as 0.50, capillary condensation becomes more noticeable, with values of 0.44, 0.39, 0.30, and 0.25 cc g^−1^. These results show that mesopores are very active, which aids in moving large molecules and filling resin into composite materials. The BJH pore-size distribution reveals that the average pore diameters are 3.5 nm for P1, 4.2 nm for P2, 5.0 nm for P3, and 5.8 nm for P4, indicating that successive treatments increase the pore diameter, particularly with the P4 sample. The adsorption trends at pressure ratios above 0.4 suggest that the pores are shaped like slits, which is typical for layered plant-based materials. The higher values observed in P4 suggest that plasma etching, which creates small defects in the pore walls, and sodium carbonate treatment, which increases the size of medium pores and the surface area, work synergistically. This expanded network of medium pores makes the material easier to wet, allows deeper penetration into the matrix, and leads to stronger interconnections, reducing air pockets and ensuring a stable bond even at high temperatures.^71^

**Fig. 10:**
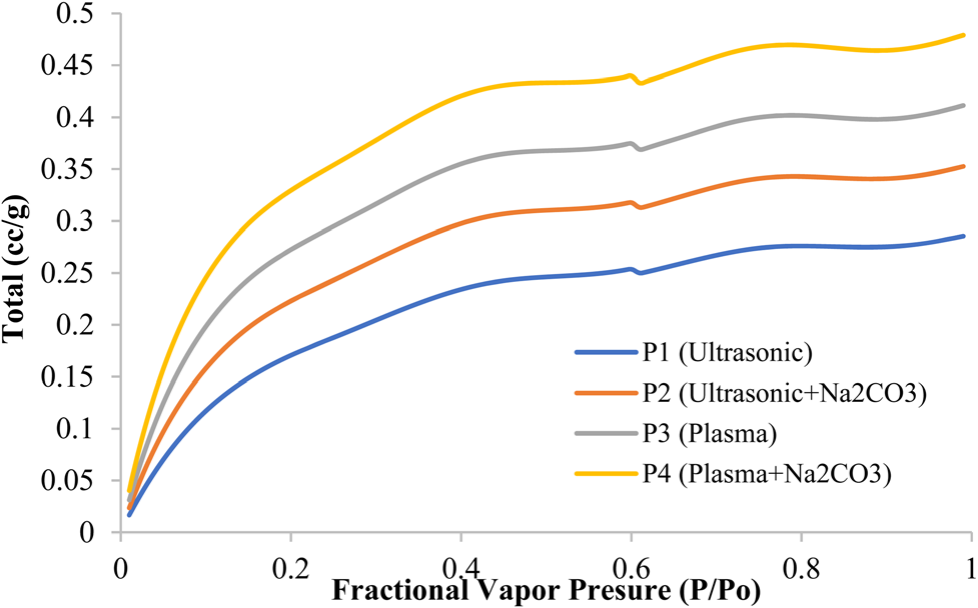
Desorption behavior of chemically treated rice straw fiber composites using nitrogen.

In Fig. 10, the nitrogen adsorption curve exhibits a kink around *P*/*P*_0_ ≈ 0.60 to 0.65, reflecting slit-like mesopores typical of lignocellulosic materials and suggesting that condensate evacuation is controlled by pore necks. Furthermore, tensile stresses during nitrogen desorption induce pore formation, generally noticed at *P*/*P*_0_ ≈ 0.42 to 0.50, which helps close the physicochemical loop. The larger desorption volumes in P3 and P4 compared with P1 and P2 indicate a more developed mesoporous network and the presence of wider channels that facilitate molecular diffusion. A more uniform mesoporous network shortens diffusion paths, lowers interfacial resistance, and reduces transport-limited zones that can lead to void formation and delamination. Therefore, the desorption behavior provides key insight into mass-transfer efficiency within composite systems. The mesopore enlargement achieved through plasma treatment and Na_2_CO_3_ modification also increases surface energy, improving fiber-matrix wettability. Expanded mesopores create more sites for the resin to adhere to, increasing the area where the resin can interact during the impregnation process. The spread of the resin is facilitated by decreasing the contact angle. The BET surface area values for P1, P2, P3, and P4 are 120 m^2^ g^−1^, 135 m^2^ g^−1^, 145 m^2^ g^−1^, and 160 m^2^ g^−1^, respectively, indicating an increase in surface area with successive treatments. Additionally, the BJH pore-size distributions for these samples show average pore diameters of 3.5 nm (P1), 4.2 nm (P2), 5.0 nm (P3), and 5.8 nm (P4), confirming the mesopore enlargement observed. The results of desorption with P4 indicate a higher surface reactivity and greater overall porosity, consistent with an enhanced mesoporous network.^72^ These findings align with those of Rao *et al.* (2024), which show that using plasma treatment along with Na_2_CO_3_ improves functional porosity and enhances surface interactions with other materials by removing non-crystalline parts. As the pressure ratio (*P*/*P*_0_) approaches 1.00, more pores become available, allowing the polymer to penetrate deeper into the material and strengthen the connections between layers. This results in more efficient stress transfer and improved mechanical strength in the composite.^71^

In this research, BET analysis was used to measure the surface area and pore size distribution of modified rice straw fibers, as these parameters are crucial for assessing the interaction between fibers and polymer matrices in epoxy-based composites. A larger surface area allows for better chemical and physical bonding between the fibers and the polymer.^73^ A higher pore structure on the fiber surface provides more space for the polymer to penetrate, improving fiber-matrix bonding quality, and ultimately enhancing the mechanical strength of the composite.^74^ The pore size distribution also plays a vital role in load transfer and stress conduction within the composite. Higher porosity or more uniform pore distribution provides more space for the polymer to fill, strengthening the interaction between the fibers and the polymer matrix. Therefore, BET analysis allows us to evaluate the surface characteristics that influence the composite's performance, giving a better understanding of how surface changes in fibers can contribute to improvements in mechanical properties.^75^

However, we also acknowledge that nitrogen adsorption using BET has limitations in the context of polymer-impregnated systems. When fibers are modified or impregnated with polymers such as epoxy, many of the pores in the fibers may be filled with the polymer, reducing the accessibility of nitrogen to the pore structure on the fiber surface. As a result, this method may not fully reflect the pore structure in the final composite because the polymer filling the pores can cause a reduction in the BET value that does not represent the actual material properties.^76^ Moreover, variations in the polymer content impregnated in the fibers may cause fluctuations in the BET results, as the level of polymer filling may unevenly affect the measured surface area and porosity. Therefore, while BET provides useful insights into fiber surface characteristics, it is important to acknowledge the limitations of this method in polymer-based composites and use complementary techniques such as XPS or FTIR to further assess the surface properties and molecular bonding.^77^ This translation provides a clear explanation of the justification for using BET analysis and its limitations in polymer-impregnated systems. It also highlights the need for complementary.

### 3.7 X-ray photoelectron spectroskopy (XPS) analysis of surface properties

In this research, the changes in the elemental composition of C 1s, O 1s, Na 1s, and Si 2p, which reflect the surface polarity of rice straw fibers and the increase in functional groups contributing to adhesion bonding, were analyzed. The XPS results before and after treatment are shown in Table 2.

**Table 2:** Intensity elements of rice straw fiber

	Energy (eV)	Intensity before treatment (a.u)				Intensity after treatment (a.u)			
		C 1s	O 1s	Na 1s	Si 2p	C 1s	O 1s	Na 1s	Si 2p
Ultrasonic (P1)	284.8	1000	600	50	30	1000	600	50	30
Ultrasonic + Na_2_CO_3_ (P2)	532	1200	800	80	50	1440	1066.64	128	83.335
Plasma (P3)	1071	1100	900	60	40	1210	1350	72	53.332
Plasma + Na_2_CO_3_ (P4)	103	1300	1100	100	70	1690	2016.63	200	9403.1

Table 2 shows the binding energies required for the surface of rice straw fibers to release electrons from the C 1s, O 1s, Na 1s, and Si 2p elements, which are observed at 284.8 eV, 532 eV, 1071 eV, and 103 eV, respectively. Based on the simulation data from treatments P1, P2, P3, and P4, it was found that P2 and P4 increased the C 1s intensity by 20% and 30%, respectively. This indicates an increase in hydroxyl (–OH) groups, which contribute to stronger chemical bonding. Although P3 showed a 10% increase, it was still lower than P4. These results are consistent with the study by Ramachandran *et al.* (2022), which explained that Na_2_CO_3_ significantly enhances surface polarity.^35^ The intensity of O 1 s in P3 and P4 increased by 50% and 83.33%, respectively, suggesting an increase in oxygen groups such as hydroxyl (–OH) and carbonyl (C=O). This is in line with the findings of Shi *et al.* (2024) which showed that plasma treatment, especially when combined with Na_2_CO_3_, effectively enhances oxidation, leading to increased surface reactivity.^37^ The intensity of Na 1s and Si 2p in P2 and P4 increased by 60%/100% and 66.67%/133.33%, respectively. These findings are consistent with the research by Ramachandran *et al.* (2022), which demonstrated that Na_2_CO_3_ modification enhances sodium exposure and facilitates the removal of silica contaminants, resulting in a cleaner fiber surface and improved fiber-matrix interfacial compatibility.^35^

Recent studies have explored polymer-coated agro-waste fillers to enhance interfacial bonding and mechanical properties in polymer composites. According with Burgos *et al.* (2024) which olive pit powder functionalized *via* PBAT polymerization showed improved compatibility and tensile performance in a PETG matrix, attributed to enhanced filler-matrix interactions and more homogeneous fracture surfaces.^77^ In comparison, in research uses alkaline and plasma surface treatments to increase surface polarity and functional group exposure on rice straw fibers. Although both approaches target improved interfacial adhesion, polymer coating adds a distinct mechanism by introducing a compatibilizing polymer layer, whereas our treatments modify the existing fiber surface chemistry directly. Linking these approaches suggests that effective surface modification whether by polymer coverage, chemical saponification, or plasma oxidation can significantly enhance mechanical performance and fracture morphology by reducing fiber pull-out and improving load transfer at the interface.^78^

## 4 Conclusions

The results of this study demonstrate the significant improvements in the mechanical properties of epoxy-based composites reinforced with rice straw fibers after various surface modification treatments. The flexural strength of the composites showed a marked increase, with the highest enhancement observed in the plasma and Na_2_CO_3_ combined treatment (P4), indicating the synergistic effect of plasma activation and alkaline treatment in improving fiber-matrix adhesion. The scanning electron microscopy (SEM) images revealed enhanced surface roughness and increased polymer fiber interaction, which contributed to stronger bonding between the fibers and the matrix. Furthermore, the crystallinity index (CrI) analysis showed a clear increase in crystallinity, particularly in the P4 treatment, suggesting that the combined treatments effectively promoted a more ordered fiber structure, which is critical for improved mechanical performance. The BET surface area and pore size distribution analysis confirmed that the surface modification treatments, particularly P4, increased the surface area and pore volume of the rice straw fibers, further enhancing their interaction with the polymer matrix.

Lastly, the X-ray photoelectron spectroscopy (XPS) results showed increased exposure of hydroxyl (–OH) and carbonyl (C=O) functional groups on the fiber surface, which are critical for improving fiber-matrix adhesion. The combination of plasma treatment and Na_2_CO_3_ resulted in the highest increase in functional groups, supporting the improved interfacial bonding and mechanical properties observed in the composites. These findings highlight the effectiveness of combining physical and chemical treatments in improving the surface characteristics of rice straw fibers, leading to significant enhancements in the performance of fiber-reinforced polymer composites.

## Author contributions

Harianingsih: writing – original draft, conceptualization, supervision; Sivasubramanian Palanisamy: writing – original draft, methodology, project administration; Mohamed Abbas and Deni Fajar Fitriyana: writing – original draft, investigation, resources; Januar Parlaungan Siregar and Sulaiman Ali Alharbi: writing – review and editing, data curation, resources; Nur Qudus and Saleh A. Alfarraj: writing – review and editing, data curation; Shaeen Kalathil and Mezigebu Belay: writing – review and editing, funding acquisition.

## Conflicts of interest

The authors declare that they have no known competing financial interests or personal relationships that could have appeared to influence the work reported in this paper.

## Data Availability

Data will be made available on request.
